# Transcriptomic and metabolomic analysis of a non-*cyp51A* mutant azole-resistant *Aspergillus fumigatus* isolated from Ningxia, China

**DOI:** 10.3389/fmicb.2025.1666905

**Published:** 2025-09-29

**Authors:** Wanting Ma, Yuting Kang, Qiujie Li, Pengtao Wang, Wei Jia

**Affiliations:** ^1^First Clinical Medical College, Ningxia Medical University, Yinchuan, Ningxia, China; ^2^Ningxia Key Laboratory of Clinical and Pathogenic Microbiology, Institute of Medical Sciences, General Hospital of Ningxia Medical University, Yinchuan, Ningxia, China; ^3^Center of Medical Laboratory, General Hospital of Ningxia Medical University, Yinchuan, Ningxia, China

**Keywords:** *Aspergillus fumigatus*, non-*cyp51A*, azole resistance, transcriptome, metabolomic analysis

## Abstract

**Background:**

Azole-resistant *A. fumigatus* (*Aspergillus fumigatus*) has been extensively documented both domestically and internationally, with mutations in the *cyp51A* gene identified as the predominant mechanism of resistance. However, the incidence of clinical non-*cyp51A* mutation-resistant *A. fumigatus* has gradually increased in recent years, and the resistance mechanisms remain unclear.

**Methods:**

We isolated a non-cyp51A mutant azole-resistant strain of *A. fumigatus*, designated Af68, from Ningxia. The fungus was further characterized using MALDI-TOF mass spectrometry and DNA sequencing of the *beta-tubulin* gene and the *calmodulin* gene for molecular characterization. We analyzed the growth diameters and responses of the azole-sensitive strain Af293 and the azole-resistant strain Af68 to various concentrations of oxidative agents, including menadione, H_2_O_2_, sodium dodecyl sulfate (SDS), and Congo Red. Transcriptome RNA sequencing was performed to identify differentially expressed genes between Af293 and Af68. Furthermore, a liquid chromatography-mass spectrometry (LC-MS) system was utilized for a comparative metabolomic analysis between the two strains. The mRNA levels of *cyp51A*, *cyp51B*, *MDR2*, *sitT*, *catA*, and *SOD2* were quantified using real-time quantitative PCR (qRT-PCR).

**Results:**

Compared to the wild-type *A. fumigatus* Af293, the strain Af68 exhibited a significantly increased growth diameter when exposed to various oxidative agents. However, no significant difference in radial growth was observed between the two strains when cultured in potato dextrose agar (PDA) medium at 37 °C on days 1 and 5. Transcriptional alterations between the two strains were analyzed using RNA-sequencing technology, revealing 594 genes with significant expression differences. The mRNA levels of C6 transcription factors, the bZIP transcription factor MeaB, and stress-activated MAP kinase interacting proteins were significantly reduced in Af68, while the mRNA levels of C2 domain-containing proteins, zinc metalloproteases, and MFS transporters were significantly increased. GO (Gene Ontology) analysis suggested that cellular processes, metabolic pathways, localization, and bioregulation collectively contribute to the biological processes underlying drug resistance. KEGG (Kyoto Encyclopedia of Genes and Genomes) enrichment analysis indicated that the differentially expressed genes are primarily associated with autophagy, amino sugar and nucleotide metabolism, ABC transporters, homologous recombination, and DNA mismatch repair. A total of 129 distinct metabolites were screened from the two groups of samples, of which 96 were found to be upregulated in the Af68. These metabolites encompass various categories, including organic acids, amino acid derivatives, peptides, natural products, and nucleotides. Furthermore, these metabolites are primarily enriched in metabolic pathways such as tyrosine metabolism, purine metabolism, D-amino acid metabolism, and glutathione metabolism.

**Conclusion:**

In conclusion, this study offers new insights and perspectives on the research of non-*cyp51A* mutation-related resistance mechanisms, utilizing phenotypic experiments, transcriptome sequencing, and metabolite analysis.

## 1 Introduction

*A. fumigatus* (*Aspergillus fumigatus*) is a widely distributed fungal pathogen, and its spores have been demonstrated to cause invasive pulmonary aspergillosis in immunocompromised patients (Bader, 2021; Earle et al., 2023). Currently, the three primary classes of first-line antifungal medications used in clinical treatment are azoles, polyenes, and echinocandins. However, the extensive use of azoles, including itraconazole, voriconazole, posaconazole, and isavuconazole, has led to the emergence of global azole resistance over the past few decades, resulting in a high mortality rate due to treatment failure in cases of aspergillosis (Pimienta et al., 2022). Common mechanisms of azole resistance in *A. fumigatus* are primarily attributed to point mutations in the *cyp51A* gene or the insertion of tandem repeat sequences (TRs) in the promoter region of *cyp51A* (e.g., TR34/L98H and TR46/Y121F/T289A). These mutations have been shown to cause alterations in sterol biosynthesis pathways (Da Fonseca et al., 2023). Furthermore, clinically resistant strains with non-*cyp51A* mutations are increasingly reported across various regions, with prevalence rates ranging from 15 to 60% (Gonzalez-Jimenez et al., 2021).

RNA-seq is a widely used method for transcriptome profiling in transcriptome analysis, employing next-generation sequencing technology (Caicedo-Montoya et al., 2019). This technology facilitates the identification of genetic differences between azole-resistant and sensitive strains. Subsequent Gene Ontology (GO) and Kyoto Encyclopedia of Genes and Genomes (KEGG) analyses can further explore functional-level alterations, elucidating the potential molecular mechanisms underlying *A. fumigatus* resistance to azoles. Furthermore, RNA-seq offers advantages such as low cost, high throughput, and high sensitivity compared to traditional sequencing methods, making it a widely adopted approach for transcriptome analysis across various species, including prokaryotes (Gong et al., 2021). Metabolomics, on the other hand, constructs metabolic networks and elucidates the mechanisms of microbial metabolite formation by analyzing differences in metabolites and metabolic pathways, thereby enhancing the interpretation of gene expression results. The joint analysis of transcriptomics and metabolomics has been explored across various fields (Tang et al., 2023); however, relatively few studies have investigated the synergistic application of both methodologies to explore metabolic pathways and key genes associated with metabolites, aiming to elucidate the mechanisms of drug resistance in fungal strains from a cause-and-effect perspective.

Our research group previously conducted drug sensitivity experiments on the *Aspergillus fumigatus* strain Af68, identified as a non-*cyp51A* mutant azole-resistant strain (Kang et al., 2024). In this study, we further examined its radial growth and antioxidant capacity. Concurrently, we employed RNA-seq technology to analyze the transcription levels of genes in both the sensitive group (Af293) and the azole-resistant group (Af68). Functional enrichment studies of the differentially expressed genes were conducted using the GO and KEGG databases. Additionally, we utilized LC–MS/MS full-spectrum metabolomics to detect metabolites in the different strains. Metabolic pathway enrichment analysis was performed through multivariate statistical analysis, including Variable Importance in Projection (VIP) value and q-value (adjusted *p*-value) screening of the differential metabolites across each group. This comprehensive analysis aimed to explore the potential resistance mechanisms of azole-resistant strain harboring a non-*cyp51A* mutation.

## 2 Materials and methods

### 2.1 Isolation and identification of *Aspergillus fumigatus* strains

The clinical *A. fumigatus* strain Af68 was isolated from the General Hospital of Ningxia Medical University, while the wild-type strain Af293 was generously provided by Professor Liu Wei from Peking University First Hospital. Both strains were inoculated on Sabouraud Dextrose Agar (SDA) and incubated at 25 °C for 1 week. Following microscopic examination, the fungus was further identified as *A. fumigatus* using the mass spectrometry system BIOMERIEUX VITEK MS, which utilizes a confidence value threshold of ≥ 60% for the identification of filamentous fungi at the species level. Subsequently, its DNA was extracted for molecular characterization through the amplification of the beta-tubulin gene (*benA,* Accession No. DQ438501.1) and the calmodulin gene (*CaM,* Accession No. LC589317.1). The resulting PCR products were sequenced and compared with reference sequences available in GenBank.

### 2.2 Radial growth assessment

The strains were incubated at 37 °C for 7 days. After incubation, the conidia were collected in sterile purified water and mixed homogeneously. The concentration of conidia was then adjusted to 1 × 10^6 conidia/ml. A volume of 2 μL of the conidia suspension was inoculated onto 10-cm plates containing Potato Dextrose Agar (PDA) medium for incubation and observation. The diameter of growth was recorded daily.

### 2.3 Stress tolerance assay

To evaluate sensitivity to oxidation and cell wall pressure, we inoculated 2 μL of a conidial suspension (1 × 10^6 conidia/mL) onto a 6-well plate containing PDA medium supplemented with various concentrations of menadione (0 μM, 30 μM, and 40 μM). Additionally, 100 μL of the same conidial suspension (1 × 10^6 conidia/mL) was spread on a PDA plate, and 20 μL of different concentrations (30, 15, 7.5, and 3.75%) of H_2_O_2_ were added to the center of the 6-well plate. Furthermore, 2 μL of a conidial suspension (1 × 10^5 conidia/mL) was spotted onto solid PDA medium in the 24-well plate supplemented with varying concentrations of sodium dodecyl sulfate (SDS) (0, 0.005, 0.01, 0.015, and 0.02%) and Congo Red (0, 50, 100, 200, 400, and 600 μg/mL), with each treatment tested independently. Each concentration was assessed separately, and the radial growth of the strain was measured after 48 h. Menadione is dissolved in DMSO, while H_2_O_2_, SDS, and Congo Red are all dissolved in ddH_2_O.

### 2.4 Transcriptome RNA sequencing

*A. fumigatus* was inoculated onto SDA plates and incubated at 25 °C for 1 week. Following incubation, the conidia were collected and suspended in sterile water to achieve a turbidity equivalent to the 0.5 McFarland standard. A 5 μL of the conidia suspension was added to 5 mL of Sabouraud broth, and the culture was shaken at 200 rpm at 37 °C for 3 days until round, spherical mycelial aggregates became visible. Subsequently, the samples were prepared for RNA extraction.

Total RNA was isolated using the E. Z. N. A.^®^ Fungal RNA Mini Kit, as per the manufacturer’s instructions (Omega, R6840). A certain amount of RNA samples are denatured at suitable temperature to open their secondary structure, and mRNA is enriched by oligo (dT) -attached magnetic beads. Subsequently, the mRNA is fragmented, and cDNA synthesis is performed. This is followed by end repair, the addition of an “A” tail, and adapter ligation. The resulting products are then amplified using a PCR reaction system. The selection of mRNA is achieved through the method of poly-A enrichment. The insert size ranges from 300 to 500 bp. After denaturation of the PCR products into single strands, they undergo DNB-based libraries circularization treatment, followed by sequencing using DNBSEQ G400 platform (BGI-Shenzhen, China).

The raw data was filtered using SOAPnuke, which involved the removal of adapter sequences, elimination of low-quality reads, and exclusion of reads with excessively high proportions of unknown bases, resulting in high-quality clean reads. The clean data were mapped to the reference genome using HISAT (v2.1.0), and the reference genome build used was Af293 assembly version: GCF 000002655.1 ASM265v1_genomic.fna.gz. The sequencing read layout was PE150, with a per-sample sequencing depth of 6G and three biological replicates per group. The differential expression (DE) method employed was DESeq2, and normalization was conducted using FPKM. Differential expression analysis was performed using DESeq2 (v1.34.0) with a q-value (FDR-adjusted p) ≤ 0.05. We then utilized RSEM to calculate the gene expression levels for each sample based on RNA-Seq data. Finally, we conducted clustering analysis on all differentially expressed genes (DEGs) between the two groups and performed functional enrichment analysis on these genes within each cluster. The transcriptome data for strains Af293 and Af68 have been submitted to the NMDC data center, passed review, and received the identifier number NMDC10019543. The total mapped reads per sample, mapping rate, and DEGs are presented in Supplementary file 1.

### 2.5 Metabolomic analysis

Conidia of *A. fumigatus* were inoculated at a concentration of 1 × 10^5 cells/mL in 15 mL of Sabouraud broth medium within 50 mL centrifuge tubes. The cultures were shaken at 200 rpm in a shaking incubator at 37 °C for 12 h. Subsequently, the cultures were centrifuged at 1000 rpm for 5 min, after which the supernatant was discarded, and the conidia were collected for detection. Each group consists of 6 samples, which are mixed in small amounts and then divided into three portions, each subjected to 2 μL for analysis. The RSD threshold is set to below 15%. Batch correction is performed using the limma package in R for data preprocessing, transformation (such as variance-stabilizing transformation), batch effect detection, and correction. Samples were analyzed using a liquid chromatography-mass spectrometry (LC–MS) system composed of the ACQUITY UPLC I-Class plus ultra-high-performance liquid chromatography and the QE plus high-resolution mass spectrometer, along with relevant databases. The LC–MS data were processed using Progenesis QI V2.3 (Nonlinear Dynamics, Newcastle, United Kingdom) software for baseline filtering, peak identification, integration, retention time correction, peak alignment, and normalization. Ionization modes include positive ion mode and negative ion mode. Principal Component Analysis (PCA) was performed to observe the overall distribution among samples and the stability of the entire analytical process. The corrected data should be validated using methods such as PCA to confirm that batch effects have been effectively eliminated. Missing-value handling involves deleting ion peaks with missing values within a group where the mean (0 values) exceeds 50%, and replacing the remaining 0 values with half of the minimum intensity of all ions across all samples. Orthogonal Partial Least Squares Discriminant Analysis (OPLS-DA) and Partial Least Squares Discriminant Analysis (PLS-DA) were employed to distinguish differences in metabolites among groups. The identification criteria for MSI levels 1–4 are as follows: Level 1: RT: +0.3 min (18 s) & Fragmentation Score ≥ 45; Level 2: RT: +0.3 min (18 s) & 0 ≤ Fragmentation Score < 45; Level 3: Fragmentation Score ≥ 45; Level 4: Fragmentation Score < 45. The Variable Importance in Projection (VIP) values obtained from the OPLS-DA model were used to rank the overall contribution of each variable to group discrimination. Differential metabolites were identified based on VIP values greater than 1.0 and q-values less than 0.05. Furthermore, pathway enrichment analysis (such as KEGG) and network analysis were conducted to elucidate the functions of the differential metabolites. The databases used include HMDB, KEGG, and LIPID MAPS. All original data and results from the metabolomics mass spectrometry analyses are presented in Supplementary file 2.

### 2.6 qRT-PCR

The mRNA levels of *cyp51A*, *cyp51B*, *MDR2*, *sitT*, *catA*, and *SOD2* were assessed using real-time quantitative PCR (qRT-PCR). In summary, conidia (1.0 × 10^7 cells/mL) were incubated at 35 °C with shaking at 200 rpm for 12 h in 5 mL of Sabouraud liquid medium. Following centrifugation, the supernatant was discarded, and the mycelia were collected for RNA isolation using the RNA simple Total RNA Kit (TIANGEN Biotech, China) in accordance with the manufacturer’s guidelines. The RNA was used as a template to synthesize complementary DNA (cDNA) using the PrimeScript RT Master Mix kit (TaKaRa Biotechnology, China), following the manufacturer’s recommendations. The qPCR process employed specific primers (Supplementary file 3) with LightCycler 480 SYBR Green I master mix (Roche). The Af293 strain was utilized as a control for susceptibility. Gene expression variation was calculated using the 2^−ΔΔCT^ method, with actin serving as the reference gene, and each sample was analyzed in triplicate.

### 2.7 Statistical analysis

All data were analyzed for normal distribution using the Shapiro–Wilk test. Differences between the two groups with multiple factors using two-way ANOVA methods conducted with GraphPad Prism 8.0. The effect sizes (eta squared), 95% confidence intervals, variance homogeneity checks (Levene’s), and exact *p*-values are reported in the Supplementary file 4.

## 3 Results

### 3.1 Radial growth of *Aspergillus fumigatus*

**Figure 1:**
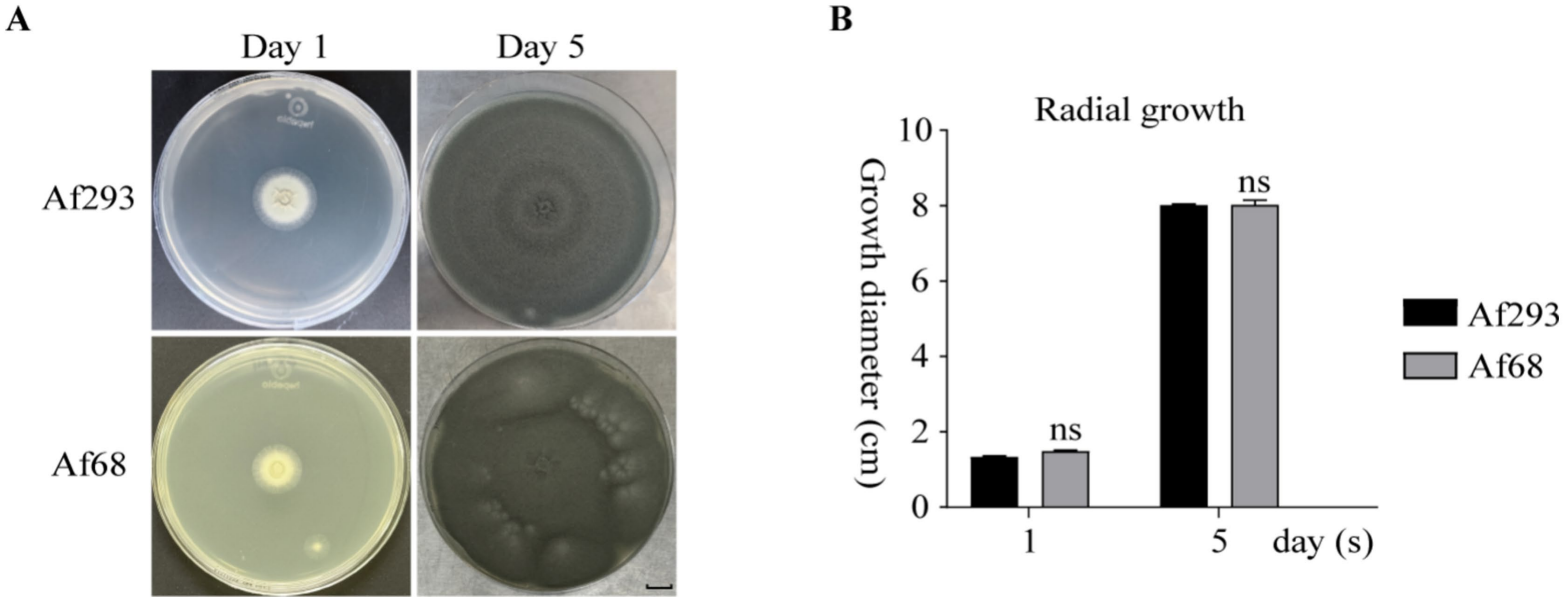
The morphological assessment of Af293 and Af68 is presented. **(A)** Morphological images of the isolates on PDA at days 1 and 5, incubated at 37 °C in a 10-cm diameter plate, are displayed. **(B)** A statistical analysis of the colony diameters at days 1 and 5 is provided. All data were obtained from three independent replicates of the experiments. The scale in the figure is 10 mm. The statistical data from all experiments are derived from independent repetitions conducted three times, presented as mean ± SD. The error bars shown in the figure represent mean + SD (ns indicates non-significance; comparisons were conducted using Sidak’s multiple comparisons test).

We identified Af68 to be *Aspergillus fumigatus* using mass spectrometry (Supplementary file 5; Figure 1A). PCR amplification of the *beta-tubulin* and *CaM* genes from Af68 was performed, followed by Sanger sequencing. Comparative analysis with the reference sequence of Af293 revealed that the homology of these two genes exceeds 99% (Supplementary file 5; Figures 1B,C). These results confirm that Af68 is classified as *Aspergillus fumigatus*. The growth of Af293 and Af68 was monitored over incubation periods of 1 day and 5 days, respectively. On the first day, both colonies exhibited yellowish and wrinkled characteristics on their reverse sides, with no significant difference in growth (Af293: 1.33 ± 0.028 (SD) vs. Af68: 1.39 ± 0.055 (SD); *n* = 3; *p* = 0.65). By the fifth day of incubation, both Af293 and Af68 demonstrated consistent radial growth and comparable spore production (Figures 1A,B).

### 3.2 Antioxidant assay

**Figure 2:**
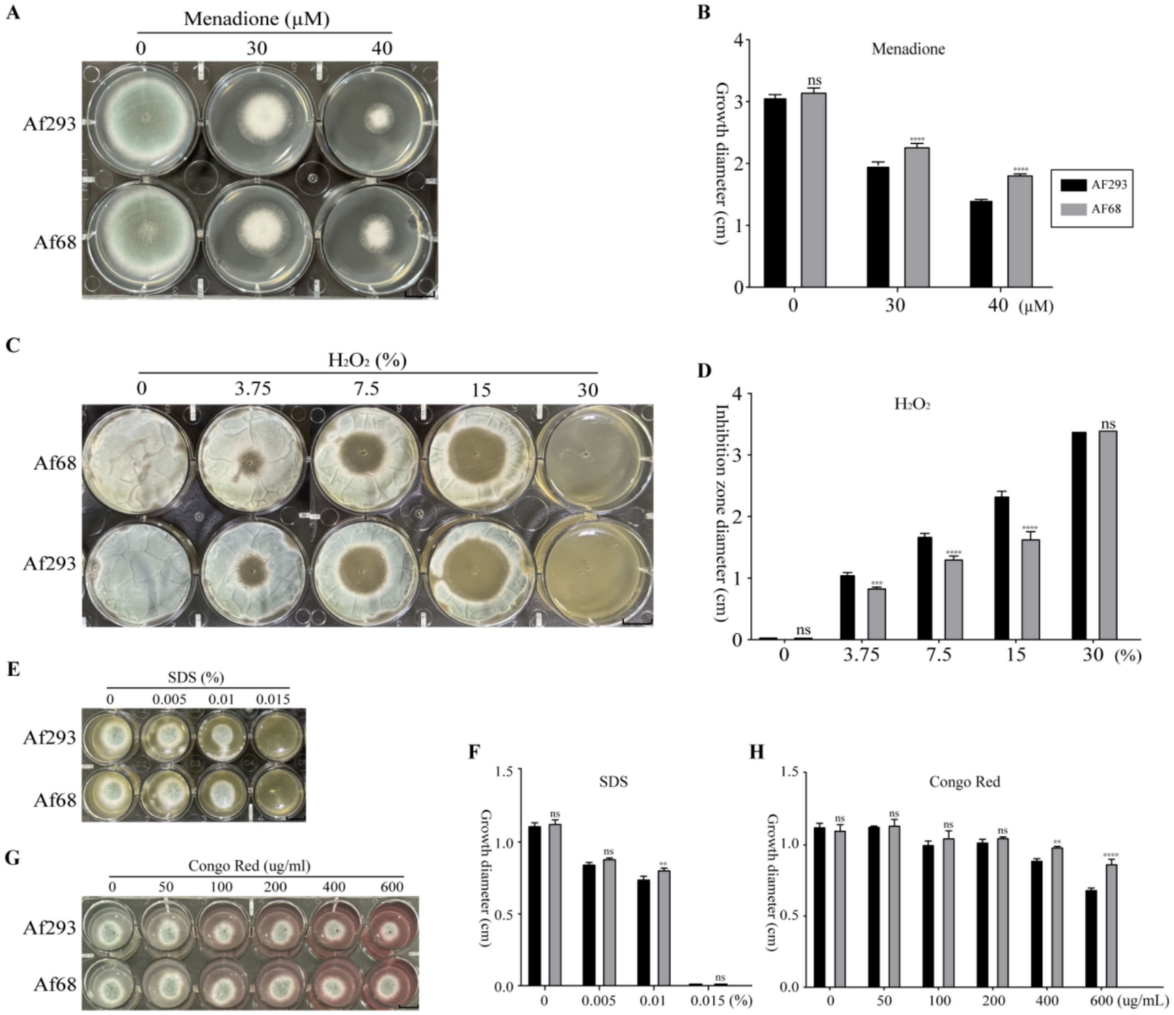
The sensitivity of *A. fumigatus* to various oxidants, including menadione, H_2_O_2_, SDS, and Congo red, was evaluated. **(A)** Morphological images of strains exposed to different concentrations of menadione (0, 30, 40 μM) are presented, accompanied by a statistical analysis of the growth diameters in **(B)**. **(C)** Morphological images of strains exposed to varying concentrations of H_2_O_2_ (0, 3.75, 7.5, 15, 30%) are displayed, alongside the statistical analysis of the inhibition zone diameters in **(D)**. **(E)** Morphological images of strains exposed to different concentrations of SDS (0, 0.005, 0.01, 0.15%) are provided, along with the statistical analysis of the growth diameters in **(F)**. **(G)** Morphological images of strains exposed to various concentrations of Congo red (0, 50, 100, 200, 400, 600 μg/mL) are presented, accompanied by the statistical analysis of the growth diameters in **(H)**. The scale in the figure is 10 mm. The statistical data from all experiments are derived from independent repetitions conducted three times, presented as mean ± SD. The error bars shown in the figure represent mean + SD (ns, non-significance; * 0.01 < *p* < 0.05; ** 0.001 < *p* < 0.01; *** 0.0001 < *p* < 0.001; **** *p* < 0.0001; comparisons were conducted using Sidak’s multiple comparisons test).

To further elucidate the differences in stress resistance between Af293 and Af68, we conducted treatments using different oxidizing agents (menadione, H_2_O_2_, SDS, Congo red). The results showed that at menadione concentrations of 30 μM and 40 μM, the growth diameter of Af68 was larger than that of Af293 (Figures 2A,B). When H_2_O_2_ was diffused from the center of the culture medium at varying concentrations (3.75, 7.5, 15%), the inhibition zone diameter for strain Af68 was smaller than that for strain Af293. Notably, at a concentration of 30% H_2_O_2_, the growth of both Af293 and Af68 was completely inhibited (Figures 2C,D). At a 0.1% concentration of SDS, the growth diameter of Af68 was greater than that of Af293; however, at other concentrations, no significant difference in growth diameter was observed between the two strains (Figures 2E,F). Under low concentrations of Congo red (50, 100, 200 μg/mL), there was no difference in growth diameter between the two groups; however, at high concentrations of Congo red (400, 600 μg/mL), the growth diameter of Af68 was greater than that of Af293 (Figures 2G,H). Despite the phenotypic differences between Af68 and Af293 under various treatments, overall, Af68 exhibited stronger antioxidant capacity than Af293.

### 3.3 Transcriptome sequencing analysis

**Figure 3:**
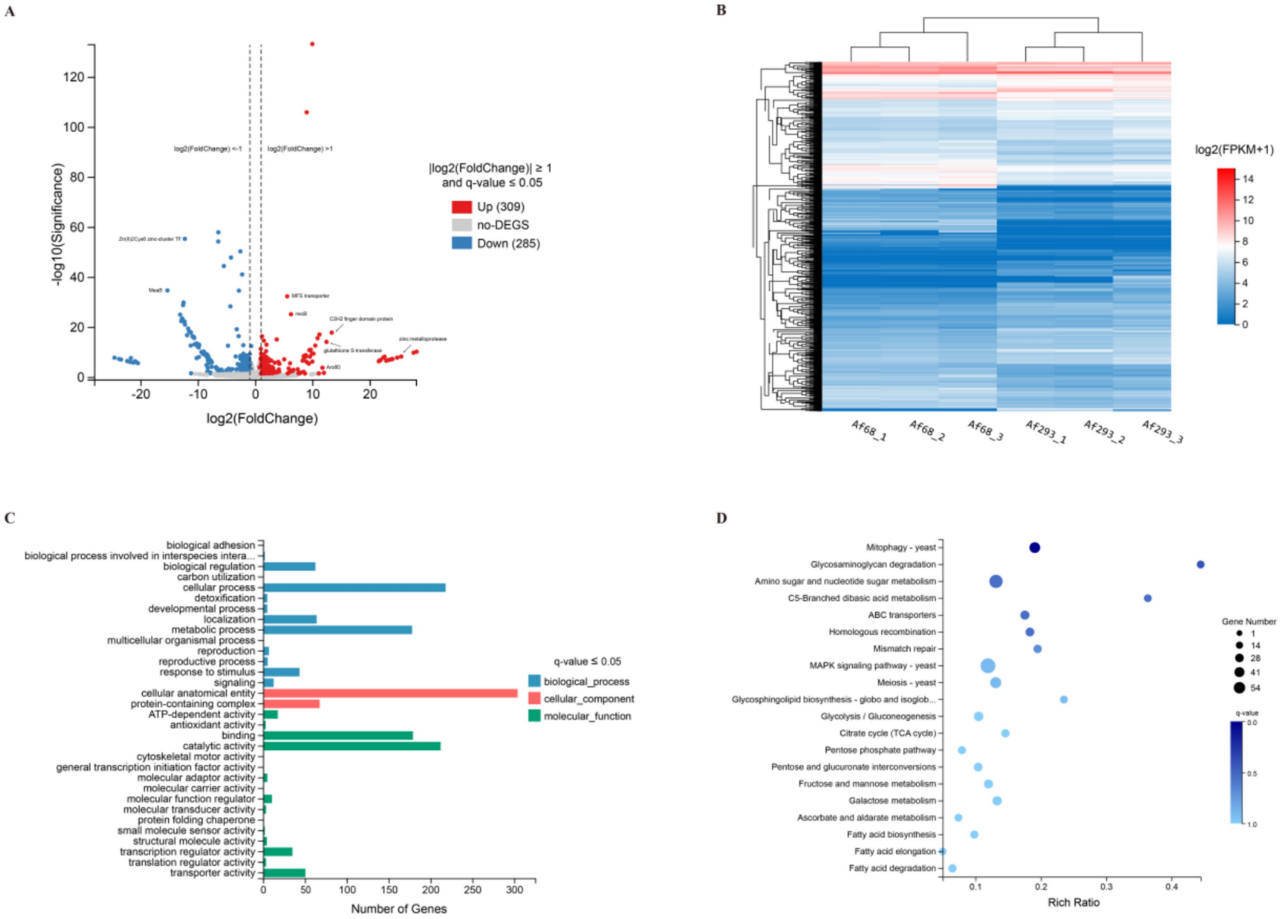
A functional analysis of differentially expressed genes in Af293 compared to Af68, utilizing various analytical methods including Volcano plot analysis **(A)**, Cluster analysis **(B)**, Gene Ontology (GO) classification **(C)**, and Kyoto Encyclopedia of Genes and Genomes (KEGG, version 114.0) analysis **(D)**.

To analyze the differences in mRNA levels between the azole-sensitive strain Af293 and the azole-resistant strain Af68, we employed transcriptome sequencing technology and conducted clustering analysis, GO, and KEGG enrichment analysis on the differentially expressed genes (DEGs). DEGs were identified based on gene expression levels in the Af293 and Af68 groups, utilizing the thresholds |log2FC| ≥ 1 and q-value ≤ 0.05. A total of 594 DEGs were identified, of which 309 were significantly up-regulated and 285 were significantly down-regulated, as illustrated in the Volcano Plot of DEGs (Figure 3A). Heatmaps in RNA-seq are utilized to depict the expression levels of genes at specific positions within the plot. Clustered heatmaps further facilitate the determination of differential expression between genes and samples. Figure 3B presents the clustering results, revealing that the Af68 group exhibited significantly lower expression levels of the Zn(II)2Cys6 zinc-cluster TF (*AFUA_2G15620*) and the bZIP transcription factor MeaB (*AFUA_3G10930*) compared to the Af293 group. Additionally, the levels of stress-activated MAPK interacting protein (*AFUA_2G12210*) and other related genes were noted. Conversely, genes encoding C2 structural domain proteins (*AFUA_7G03800*), zinc metalloprotease (*AFUA_1G12640*), and MFS transporter protein (*AFUA_3G02610*) exhibited significantly higher levels.

GO annotations were categorized into three primary groups: biological processes, cellular components, and molecular functions. The results revealed ten functional entries for biological processes, two for cellular components, and twelve for molecular functions. Representatives of differential genes associated with cellular components include cellular anatomical entities and protein complexes. In the molecular function category, these differential genes primarily participate in binding (180 genes), catalysis (213 genes), and transport (51 genes) (Figure 3C). The KEGG pathway enrichment analysis indicates that the top 20 most enriched pathways predominantly encompass autophagy, glycosaminoglycan degradation, amino sugar and nucleotide sugar metabolism, C5-branched dibasic acid metabolism, ABC transporters, homologous recombination, and mismatch repair. Notably, the MAPK signaling pathway exhibited the highest number of differentially expressed genes, followed closely by the amino sugar and nucleotide sugar metabolism pathway (Figure 3D).

### 3.4 Metabolic analysis of *Aspergillus fumigatus*

**Figure 4:**
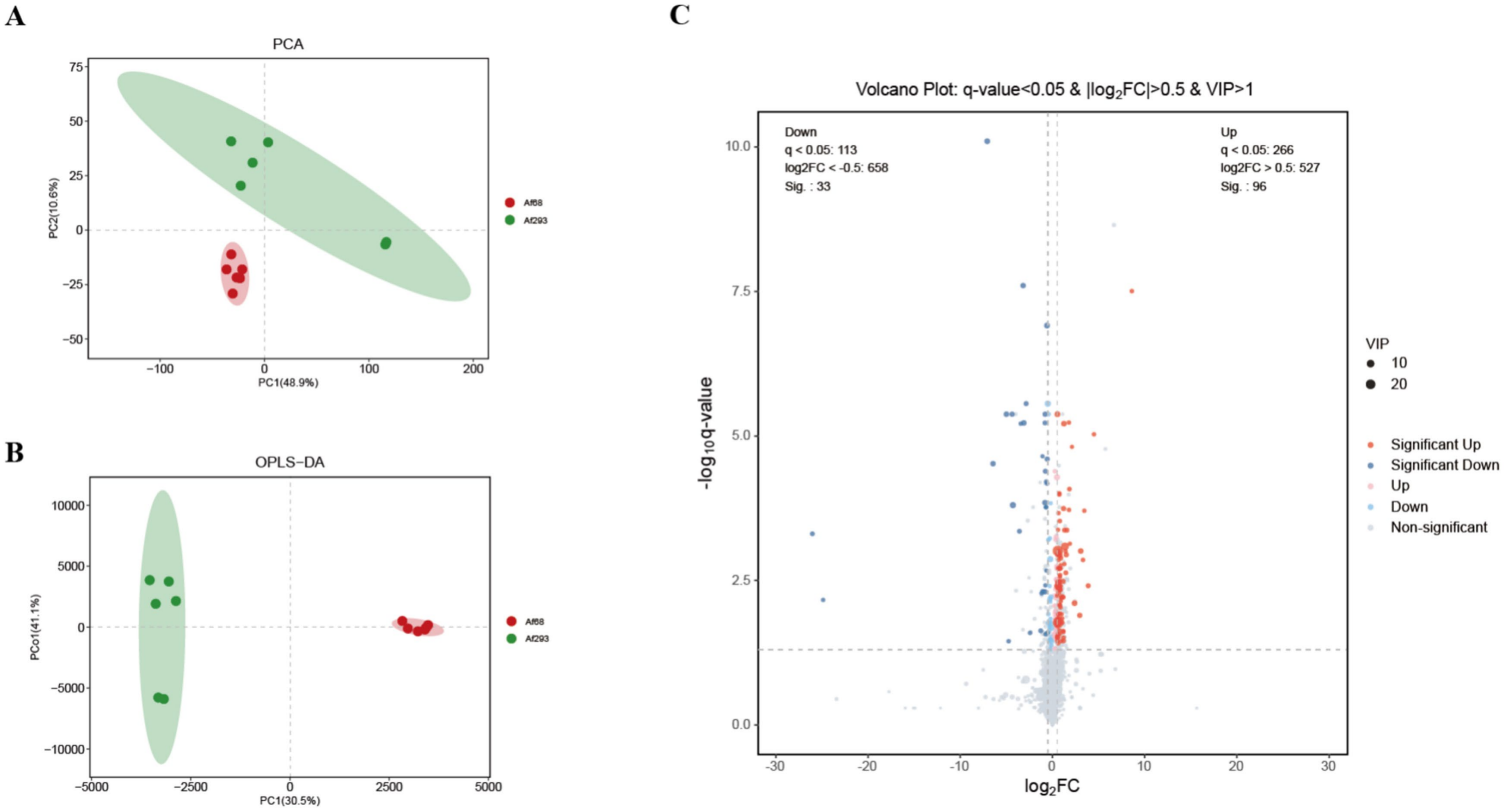
The overall differential analysis between samples Af293 and Af68 includes Principal Component Analysis (PCA:48.9% variance of PC1 and 10.6% variance of PC2) **(A)**, Cross-skewed least squares discriminant analysis (OPLS-DA: R2X = 0.795, R2Y = 0.995, Q2 = 0.917) **(B)**, and the analysis of differentially expressed metabolites between Af293 and Af68 using a volcano plot **(C)**.

To better assess the impact of non-*cyp51A* mutation on the metabolites of resistant strains, we employed liquid chromatography-mass spectrometry (LC–MS) to elucidate the variations in intracellular metabolic patterns among various strains. Principal Component Analysis (PCA) demonstrates that samples within the same group exhibit minimal differences, whereas significant differences are observed between different groups (Figure 4A), a trend that is further emphasized in the Orthogonal Partial Least Squares Discriminant Analysis (OPLS-DA) score plot (Figure 4B). In the OPLS-DA model, the diagnostic parameters R2X, R2Y, and Q2 are 0.795, 0.995, and 0.917, respectively, indicating strong performance on the training data. To mitigate the risk of model overfitting, we employed 7-fold cross-validation and 200 response permutation testing (RPT) to evaluate model quality. The results reveal R2 = 0.953 and Q2 = −0.229, suggesting that the model is not overfitting and possesses good predictive capability. Additionally, the variable importance in projection (VIP) values for various metabolites exceed 1, indicating their statistical significance. A total of 129 metabolites with significant inter-group differences were identified through the combination of multidimensional and unidimensional analysis methods (Figure 4C). Among these, 96 metabolites were upregulated (q-value < 0.05 and log_2_FC > 0.5), while 33 metabolites were downregulated (q-value < 0.05 and log_2_FC < −0.5). Compared to the sensitive group, the resistant group exhibited significant upregulation of Cinnamic Acid, N-(1-Deoxy-1-Fructosyl) Phenylalanine, Acetyl-Ser-Asp-Lys-Pro, Arginylglycine, Glycyl-Prolyl-Glutamic Acid, Desciclovir, and Inosine; conversely, Hydroxyprolyl-Proline, Macamide, and Cytidylyl−(3′,5′) − Guanosine were significantly downregulated.

### 3.5 KEGG analysis of differential metabolites

**Figure 5:**
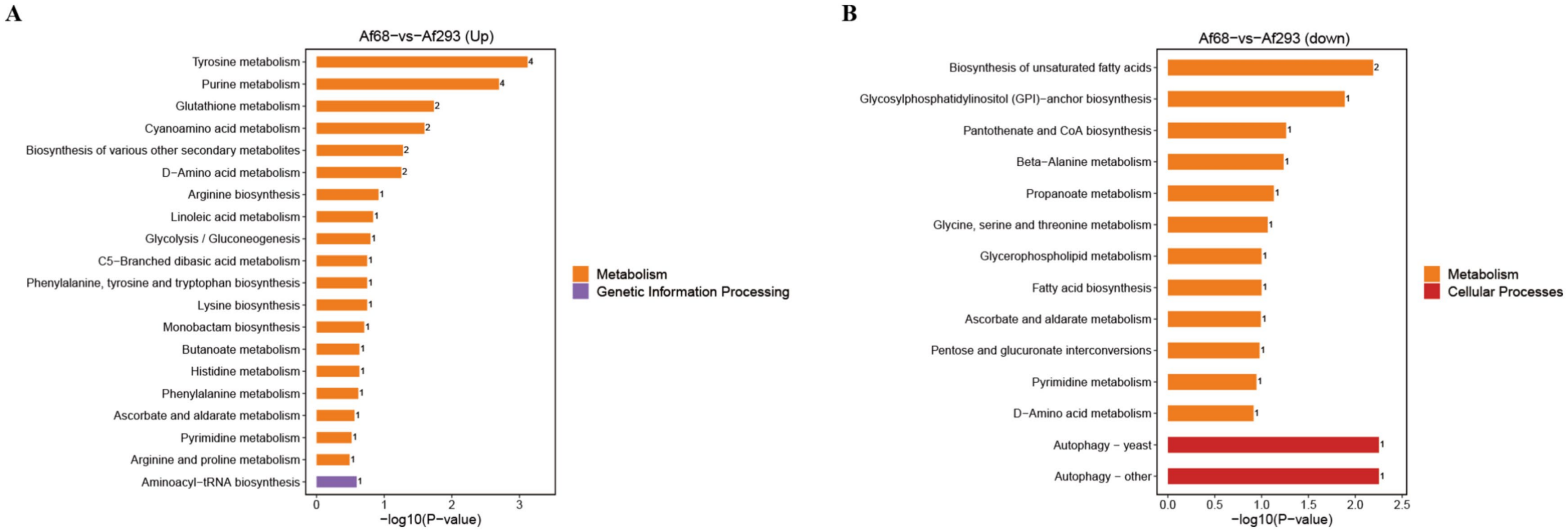
The KEGG terms were identified by applying hypergeometric tests to find pathway entries that are significantly enriched among differentially expressed metabolites compared to the entire background. The up-regulated pathways **(A)** and down-regulated pathways **(B)** of metabolites are illustrated.

We conducted pathway enrichment analysis using the KEGG IDs of differential metabolites to obtain the results of metabolic pathway enrichment. By applying hypergeometric tests, we identified pathway entries that are significantly enriched among differentially expressed metabolites compared to the entire background. Results revealed that the up-regulated metabolites were primarily associated with pathways related to tyrosine metabolism, purine metabolism, glutathione metabolism, cyanoamino acid metabolism, the biosynthesis of various secondary metabolites, D-amino acid metabolism, and aminoacyl-tRNA biosynthesis pathways involved in genetic information processing (Figure 5A). In contrast, the down-regulated metabolites were linked to pathways related to biosynthesis of unsaturated fatty acids; glycosylphosphatidylinositol (GPI)-anchor biosynthesis; pantothenate and CoA biosynthesis; beta-alanine metabolism, propanoate metabolism; glycine, serine and threonine metabolism; and glycerophospholipid metabolism (Figure 5B).

## 4 Discussion

A systematic review indicates that over 6.55 million people worldwide are threatened by invasive fungal diseases each year, resulting in 3.8 million deaths. The mortality rate of invasive aspergillosis can reach 85.2% among patients with chronic obstructive pulmonary disease, lung cancer, hematological malignancies, and those in intensive care units (Denning, 2024). Invasive aspergillus infections are primarily caused by *Aspergillus fumigatus* (Wichmann et al., 2025). Azoles (itraconazole, posaconazole, voriconazole, etc.) serve as first-line therapeutic agents, exerting antifungal effects by inhibiting ergosterol synthesis through the blockade of lanosterol 14-alpha-demethylase (*cyp51A*). However, the issue of drug resistance continues to escalate (Xiong et al., 2023; Lockhart et al., 2023). While most azole-resistant strains are linked to mutations in the *cyp51A* gene (Hsu et al., 2022), some strains demonstrate resistance or reduced susceptibility to one or more azoles without mutations in this gene, indicating the presence of non-*cyp51A* mutation-mediated resistance mechanisms. Existing studies suggest that factors such as defective mitochondrial function (e.g., mutations in the *cox10* gene), activation of drug efflux pumps, and abnormalities in the cellular stress response pathway may contribute to this type of resistance (Wei et al., 2016). For instance, Sharma et al. (2019) found that two drug-resistant strains exhibited mutations in the *cox10* gene, specifically P17S and A423V. Nonetheless, research on non-*cyp51A* drug resistance mechanisms remains in its preliminary stages, and the molecular regulatory network and key effectors have yet to be elucidated. This area requires in-depth analysis through multi-omics joint analysis and functional validation.

The group previously screened a non-*cyp51A* mutant resistant strain, observing no significant difference in radial growth between the sensitive strain Af293 and azole resistant strain Af68 during culture. In the presence of various oxidants, Af68 exhibited a significantly greater growth diameter compared to Af293, which may be attributed to the elevated mRNA levels of the oxidases *catA* and *sod2* in Af68 (Supplementary Figure 2). However, transcriptomic results indicate no differential expression of these two genes between the two groups, which may be attributed to discrepancies between the transcript samples and the qPCR samples. This capacity for stress resistance is crucial for the pathogen’s long-term survival under adverse conditions, including osmotic or oxidative inhibition, extreme growth temperatures, and nutrient limitation, and it contributes to its resistance to antifungal therapy (Zhai et al., 2021). Prolonged treatment with azoles results in the production of reactive oxygen species (ROS), which inhibit the growth of *A. fumigatus*. Therefore, adaptation to oxidative stress has been linked to azole resistance (Pérez-Cantero et al., 2020).

Among the up-regulated DEGs, the zinc metalloprotease (*AFUA_1G12640*) has emerged as a prominent research focus. Previous studies have indicated that zinc plays a crucial role in biological systems, with approximately 40% of zinc-binding proteins (Zn^2+^) functioning as transcription factors. Additionally, zinc deficiency significantly impacts various metabolic pathways. In Aspergillus, only three genes encoding Zrt-/Irt-like proteins (ZIPs) involved in zinc uptake have been characterized: *ZrfA*, *ZrfB*, and *ZrfC* (Garstka et al., 2024). Once Zn^2+^ enters the cytoplasm, it is utilized in numerous metabolic processes, including endoplasmic reticulum function, oxidative stress resistance, protein folding and synthesis, vesicle transport, and chromatin modification (Perez-Cuesta et al., 2021). This study found that the expression of zinc metalloprotease-related genes was elevated in Af68, suggesting their potential involvement in the mechanism of drug resistance associated with non-*cyp51A* mutation.

ABC transporter proteins, a class of transmembrane proteins, play a crucial role in various biological processes, including nutrient transport, drug efflux, and cellular membrane homeostasis. Certain ABC transporter proteins are notably linked to drug resistance in clinical settings. Our analysis revealed that two ABC drug transporter proteins, mdr2 and sitT, encoded by *AFUA_4G10000* and *AFUA_3G03430*, exhibited higher mRNA expression levels in Af68 compared to Af293 (Supplementary Figure 2), aligning with the transcriptomic results. Although Af68 does not possess a mutation in the *cyp51A* gene, qPCR results indicate that its mRNA levels are elevated compared to those of Af293, while no differences in *cyp51B* expression were observed. This observation suggests the potential involvement of other transcription factors or proteins in the regulation of *cyp51A* expression, such as the fungal-specific transcription factor AtrR (Hagiwara et al., 2017) and the sterol regulatory element-binding protein gene *sreA* (Liu et al., 2015). Further experiments are necessary to validate these findings. Major facilitator superfamily (MFS) multidrug transporter proteins were significantly upregulated in the azole-resistant strain, indicating that the overexpression of these efflux pumps is closely associated with drug resistance and may be activated through metabolic regulatory mechanisms (Long et al., 2021). The investigation of these proteins is advantageous for the advancement of carrier systems aimed at regulating drug delivery within cells (Li et al., 2018). These results demonstrate that *A. fumigatus* evolves various resistance strategies and molecular mechanisms for survival against drugs.

Cellular metabolism and metabolic regulation (including organic acids, amino acid derivatives, peptides, natural products, and nucleotides) play crucial roles in elucidating the mechanisms underlying the formation of drug resistance. Previous studies have confirmed that amino acid metabolism is elevated during the development of *Candida albicans* biofilms (Lindsay et al., 2024). In the present study, amino acid metabolites were found to be significantly upregulated in non-*cyp51A* mutation-resistant strains, such as glycyl-prolyl-glutamate. Although this compound has not been directly implicated in sphingolipid biosynthesis, its constituent amino acids (glycine, proline, and glutamate) may influence sphingolipid synthesis through various indirect mechanisms. Sphingolipids are essential components of the cell membrane in opportunistic fungal pathogens, and alterations in membrane composition can impact drug recognition and the activity of membrane azole efflux pumps, thereby affecting virulence and antifungal drug resistance. Previous research has demonstrated that azole resistance in *Candida* species is typically mediated by the expression of efflux pumps (Prasad et al., 2016). Consequently, Dudakova et al. proposed that the upregulation of drug efflux pumps may represent a secondary mechanism of azole resistance in *A. fumigatus* (Robbins et al., 2017). Furthermore, increased expression of centralized efflux pumps has been observed in azole-resistant clinical isolates of *A. fumigatus* with non-*cyp51A* mutation, which enhances drug efflux and reduces intracellular drug concentrations, ultimately leading to increased drug resistance.

Studies have demonstrated that the upregulation of glutathione metabolism is closely linked to azole resistance in *A. fumigatus*. Glutathione serves as a crucial antioxidant that scavenges intracellular reactive oxygen species (ROS) through various mechanisms, including modulation of the oxidative stress response, enhancement of antioxidant capacity, and influence on cell wall synthesis. This process alleviates oxidative stress induced by antifungal drugs, thereby enhancing the drug tolerance of *A. fumigatus* (Chen et al., 2024). Notably, the role of this metabolic pathway in mediating drug resistance by enhancing oxidative stress resistance in *A. fumigatus* has been established through previous phenotyping experiments, which provide a clearer illustration of the underlying mechanisms of drug resistance (Tian et al., 2024). Analysis of the upregulated metabolic pathways indicates that tyrosine metabolism in *A. fumigatus* has been less extensively studied in direct relation to drug resistance. For instance, phenolic compounds resulting from tyrosine metabolism may participate in cell wall modification, thereby affecting the penetration of antifungal drugs (Zacchino et al., 2017). The identification of differential metabolites through metabolomics analysis is essential for the rapid detection of resistant pathogens (Zhang et al., 2025).

However, this study has several limitations that warrant attention. First, the DEGs identified from the transcriptome require further validation through quantitative PCR (qPCR). Second, there is an observed lack of correlation between the DEGs and various metabolic pathways. We hypothesize the following reasons for this observation: (1) mRNA is translated into proteins, and only a limited number of metabolites are derived from proteins. Therefore, it is essential to conduct a joint analysis of transcriptomic, metabolomic and proteomic data in future studies. (2) The DEGs identified from the transcriptome may overlook certain genes, such as *cyp51A, catA*, and *sod2*, which necessitates subsequent validation to confirm their relevance. Consequently, more precise methodologies are required to accurately determine DEGs for a comprehensive analysis of metabolic pathways.

## 5 Conclusion

In summary, this study compared the radial growth, oxidative stress resistance, differentially expressed gene functions and pathways, as well as differential metabolites and metabolic pathways between the wild-type strain and the non-*cyp51A* mutant azole-resistant *A. fumigatus*. Through phenotyping experiments, transcriptome sequencing, and metabolomics analyses, this research provides a solid foundation for subsequent investigations into the resistance mechanisms associated with the non-*cyp51A* mutation.

## Data Availability

The transcriptomic data generated in this study have been submitted to the National Microbiology Data Center (https://nmdc.cn/: accession no. NMDC10019543), and the metabolomic data have been deposited in OMIX, China National Center for Bioinformation/Beijing Institute of Genomics, Chinese Academy of Sciences (https://ngdc.cncb.ac.cn/omix: accession no. OMIX010598).
