# Supplementary material for: Transcriptomic and metabolomic analysis of a non-cyp51A mutant azole-resistant Aspergillus fumigatus isolated from Ningxia, China
Source: Front Microbiol. 2025 Sep 29;16:1666905. doi: 10.3389/fmicb.2025.1666905 (PMC12516256; doi:10.3389/fmicb.2025.1666905)
Supplement: Supplementary file 5 [file Data_Sheet_5.DOCX]

**Supplementary Materials**

**
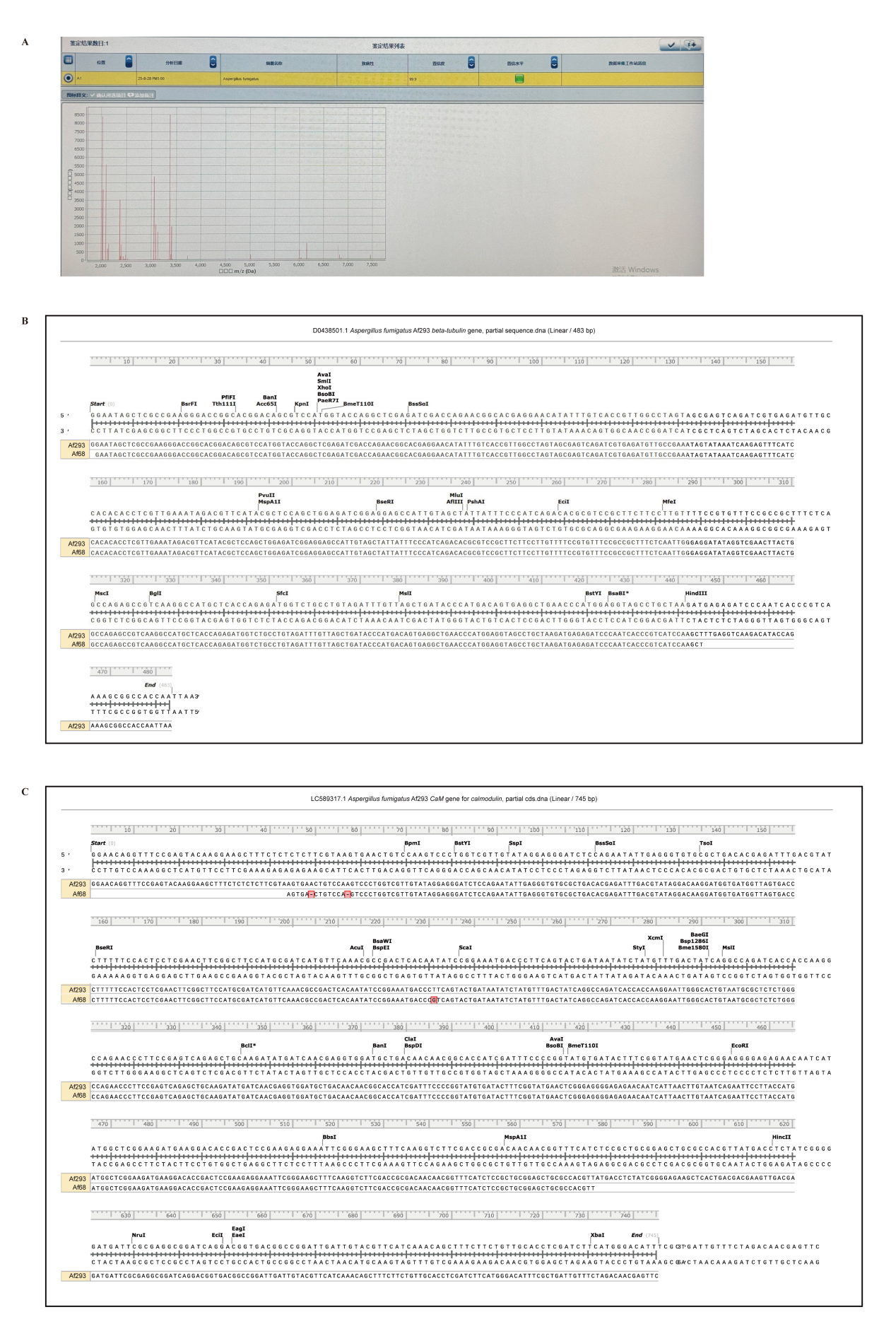
**

**Figure 1.** Mass spectrometry and PCR sequencing methods were employed to identify Af68 as *Aspergillus fumigatus*. (A) The results of mass spectrometry identification for Af68. (B) The *beta-tubulin* sequencing results for Af68 were compared with those of Af293. (C) The *CaM* sequencing results for Af68 were compared with those of Af293.


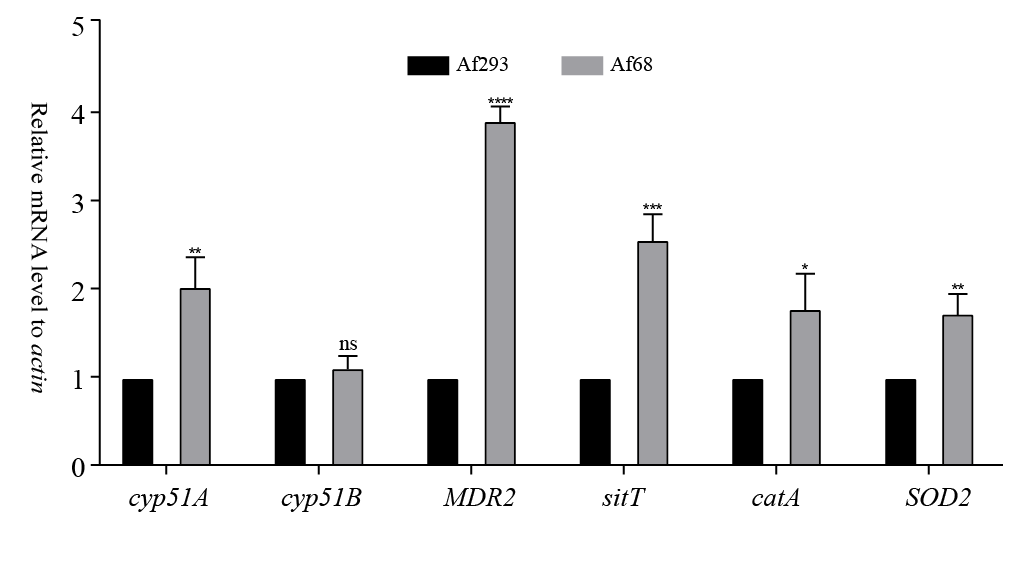


**Figure 2.** The mRNA levels of drug resistance and oxidative stress-related genes (*cyp51A, cyp51B, MDR2, sitT, catA, SOD2*) were assessed through qPCR experiments to evaluate the differences between Af293 and Af68. Actin was utilized as the reference gene. The statistical data were obtained from three independent experimental replicates. Differences between groups were analyzed using unpaired t-test methods performed with GraphPad Prism 8.0 (ns, non-significance; *0.01 < p < 0.05; **0.001 < p < 0.01; ***0.0001 < p < 0.001; **** p < 0.0001).
